# Comparison of CP-PC-SAFT and SAFT-VR-Mie in Predicting Phase Equilibria of Binary Systems Comprising Gases and 1-Alkyl-3-methylimidazolium Ionic Liquids

**DOI:** 10.3390/molecules26216621

**Published:** 2021-11-01

**Authors:** Asaf Chiko, Ilya Polishuk, Esteban Cea-Klapp, José Matías Garrido

**Affiliations:** 1 Department of Chemical Engineering, Ariel University, Ariel 40700, Israel; asafck9@gmail.com; 2 Departamento de Ingeniería Química, Universidad de Concepción, Concepción 4070386, Chile; estebancea-klapp@udeq.cl

**Keywords:** predictive modeling, SAFT, ionic liquids

## Abstract

This study compares performances of the Critical Point-based revision of Perturbed-Chain SAFT (CP-PC-SAFT) and the SAFT of Variable Range and Mie Potential (SAFT-VR-Mie) in predicting the available data on VLE, LLVE, critical loci and saturated phase densities of systems comprising CO, O_2_, CH_4_, H_2_S, SO_2_, propane, the refrigerants R22, R23, R114, R124, R125, R125, R134a, and R1234ze(E) and ionic liquids (ILs) with 1-alkyl-3-methylimidazolium ([C_n_mim]^+^) cations and bis(trifluoromethanesulfonyl)imide ([NTf_2_]^−^), tetrafluoroborate ([BF_4_]^−^) and hexafluorophosphate ([PF_6_]^−^) anions. Both models were implemented in the entirely predictive manner with *k*_12_ = 0. The fundamental Global Phase Diagram considerations of the IL systems are discussed. It is demonstrated that despite a number of quantitative inaccuracies, both models are capable of reproducing the regularities characteristic for the considered systems, which makes them suitable for preliminary estimation of selectivity of the ILs in separating various gases.

## 1. Introduction

Ionic liquids (ILs) are organic salts whose melting points are below 100 °C. Due to their negligible vapor pressures, ILs are considered as green solvents. Moreover, the selectivity of ILs in separating various compounds, including gases, attach them by significant practical importance. A number of possible combinations between cations and anions of ILs is practically unlimited. Such a variety of systems cannot be investigated experimentally. Besides that, purities of laboratory samples are typically much better than of industrial solvents, which can result in unignorable difference between literature data and actual phase equilibria in industrial equipment. Moreover, due to the high viscosities of ILs, there is no guarantee that their real solvent capacities in industrial equipment always reach equilibria values. At times, there are noticeable deviations between different laboratory measurements of phase equilibria in the same ILs systems.

These reasons emphasize a primary importance of developing and validating thermodynamic approaches capable of estimating phase equilibria and other properties of mixtures comprising ILs with reasonable accuracy in the entirely predictive manner. In this respect, it should be emphasized that the thermodynamic properties of real fluids are influenced by various molecular phenomena, which currently cannot be appropriately treated, even by the most advanced theoretical approaches. Moreover, their application in systems of non-ordinary fluids such as ILs present a particularly challenging test. Bearing in mind a potentially high uncertainty of the available experimental data on ILs systems used for validation of thermodynamic models, it seems expedient to place major attention to their overall robustness and predictive capacity rather than to the precision in local fitting of particular datasets.

Implementations of various thermodynamic approaches for modeling phase behavior in the IL systems were scored by several comprehensive reviews, see, for example [1,2,3]. Among the predictive approaches, the Conductor-Like Screening Model for Real Solvents (COSMO-RS) [4,5,6,7,8] and the Universal Quasichemical Functional-group Activity Coefficients model extended to ILs (UNIFAC-IL) [9] should be acknowledged. These models aim at estimating phase equilibria rather other thermodynamic properties, such as densities, heat capacities and sound velocities. Comparing to them, the equation of state (EoS) approaches relating pressure to temperature and volume have a more universal character. Thus far, numerous applications of Cubic EoSs (see for example [10,11,12,13,14,15,16,17,18,19,20,21,22,23,24,25,26,27]) and various versions of models belonging to a more theoretically advanced family of Statistical Association Fluid Theory (SAFT) (see for example [28,29,30,31,32,33,34,35,36,37]) for describing systems of ILs were reported in the literature. Most of these contributions have fitted the EoS approaches to the available data on mixtures using binary adjustable parameters. Although such practice substantially improves an agreement between the experimental and calculated data, it affects the predictive character of the models and hinders evaluation of their overall robustness and reliability. At the same time, some studies [38,39,40,41,42] have applied the EoS approaches to the systems of ILs in the predictive manner, while keeping zero or universal substance-independent values of the binary parameter *k*_12_. The basic principle of EoS models is utilizing an input of available experimental data to produce an output of predicted data. As the input required by any particular EoS is smaller and the obtained output is larger, its predictive value becomes higher. Accuracies of the Critical Point-based revision of Perturbed-Chain SAFT (CP-PC-SAFT) [43] and the SAFT of Variable Range and Mie Potential (SAFT-VR-Mie) [44] in estimating the elevated pressure densities and auxiliary thermodynamic properties attach these models by a particularly high degree of universality.

This study examines the performances of CP-PC-SAFT and SAFT-VR-Mie in their maximal possible level of predictivity. For each model, the input information contains only three pure compound data or six data per binary system, without any preliminary consideration of the binary data. Basing on this small amount of the input information, the models are applied for predicting vapor–liquid equilibria (VLE), liquid–liquid–vapor equilibria (LLVE), critical loci and saturated phase densities of systems comprising several industrially important gases and the ILs with 1-alkyl-3-methylimidazolium ([C_n_mim]^+^) cations (*n* = 2,4,6,8) and bis(trifluoromethanesulfonyl)imide ([NTf_2_]^−^), tetrafluoroborate ([BF_4_]^−^) and hexafluorophosphate ([PF_6_]^−^) anions. 

## 2. Theory

Full descriptions of CP-PC-SAFT and SAFT-VR-Mie are available elsewhere [43,44]. Therefore, here, some of their most relevant details are briefly discussed. Generally speaking, SAFT models express the residual Helmholtz energy *A*^res^ as a sum of contributions representing different inter-molecular interactions as functions of temperature (*T*) and molar volume (*v*). Having an expression for *A*^res^, the EoS relating pressure (*P*) with *T* and *v* can be obtained:

(1) $$P=\frac{RT}{v}-{\left(\frac{\partial A^{res}}{\partial v}\right)}_{T}$$

Several SAFT approaches, among them the initial version of PC-SAFT [45], exhibit certain undesired numerical pitfalls, such as predicting negative heat capacities [46] at high pressures and the additional unrealistic pure compound phase splits [47,48]. The latter phenomenon may seriously affect their accuracies in predicting properties of heavy compounds such as ILs and their mixtures [49,50,51]. In addition, SAFT models typically cannot represent critical pressures, temperatures, and saturated liquid densities in a simultaneously accurate manner [52,53]. To solve all these problems, CP-PC-SAFT [43] majorly revised the initial version of PC-SAFT [45]. This revision allows a standardized numerical solution of three molecular parameters, namely *m* (the effective number of segments), *σ* (the segment diameter, Å), and $\varepsilon /k_{B}$ (the potential depth divided by Boltzmann constant, K) at critical temperature (*T_c_*), critical pressure (*P_c_*) and the triple point liquid density, applying the critical point conditions as follows:

(2) $${{\left(\frac{\partial P}{\partial v}\right)}_{Tc}={\left(\frac{\partial P^{2}}{\partial^{2}v}\right)}_{Tc}=0|}_{v_{c,EoS}=\delta v_{c}}$$

(3) $$P_{c,EoS}=P_{c}$$

(4) $${\rho_{L,EoS}=\rho_{L,\mathrm{experimental}}|}_{Triple^{}Point}$$

In Equation (2), *δ* is a substance-dependent critical volume displacement of the model and it is the fourth variable obtained by solving a system of four Equations (2)–(4). Thus, in the cases of compounds considered as non-associative and non-polar, the model requires only three pure compound data, namely the literature values of *T_c_*, *P_c_*, and the triple point liquid density. In the current study, this information was obtained from the DIPPR databank [54].

Compared with CP-PC-SAFT, SAFT-VR-Mie is a more complicated approach, whose theoretical background is substantially more advanced. In addition to the three ordinary molecular parameters *m*, *σ*, and $\varepsilon /k_{B}$, it also includes *λ*_a_ (the attractive exponent of Mie potential), which is typically set to 6, and *λ*_r_ (the repulsive exponent of Mie potential). Mejía et al. [55] proposed a set of particularly successful corresponding state (CS) correlations for the molecular parameters of SAFT-VR-Mie. Similarly to CP-PC-SAFT, this method requires three pure compound data, namely *T_c_*, liquid density at *T*/*T_c_* = 0.7 and the acentric factor (*ω*). However, unlike CP-PC-SAFT, which rigorously obeys the literature values of both *T_c_* and *P_c_*, it fits only *T_c_* and overestimates *P_c_*. Table 1 lists the values of molecular parameters of the considered gases obtained by solving Equations (2)–(4) for CP-PC-SAFT and yielded by the CS approach of Mejía et al. [55] for SAFT-VR-Mie. The parameters of refrigerants were reported previously [56]. Overestimations of the literature [54] values of *P_c_* (Δ*P* = *P*_*c*,*SAFT-VR-Mie*_/*P*_*c*,*DIPPR*_) by CS-SAFT-VR-Mie are also provided. As seen, in some cases, such as R23 and R134a, these overestimations are significant. Apparently, treatment of these compounds as non-polar and non-associative is an oversimplification. However, such an approach allows preserving a predictive parametrization of the model.

Unfortunately, the genuine critical constants of ILs are unavailable because these compounds decompose at the much lower temperatures. However, the molecular parameters of SAFT models can be obtained by solving them at three standardly selected density points, two at the lowest available isotherm and one at the highest one. The resulting values for the considered ILs, along with the predicted values of *T_c_* and *P_c_*, are listed in Table 2. The CP-PC-SAFT parameters of ILs belonging to the [C_n_mim][NTf_2_] homologies series were generalized by molecular weight (*M*_w_) [39]. Following the Lennard–Jones potential, the SAFT-VR-Mie parameters *λ*_a_ and *λ*_r_ were set to 6 and 12. The estimations of densities in wide range of *T* and *P* yielded by both models are particularly accurate, and the deviations from data are typically comparable with experimental uncertainties [39,42]. As seen, SAFT-VR-Mie yields the higher values of *T_c_* and *P_c_* versus CP-PC-SAFT.

## 3. Results and Discussion

As discussed [41,42], the pressure dependencies of densities of the considered ILs decrease with reducing the *n*-alkyl chain of [C_n_mim]^+^ cation and lessening the anion’s size from [NTf_2_]^−^ to [PF_6_]^−^ and then to [BF_4_]^−^. It can also be seen (Table 2) that the imaginary *T_c_* and *P_c_* values of ILs generated by CP-PC-SAFT increase with decrease of the pressure dependencies. SAFT-VR-Mie partially reproduces this inter-relation as well. According to the general Global Phase Diagram (GPD) considerations, as the gap between the critical points of solutes and solvents decrease, the systems become more symmetric, their phase splits become narrower, resulting thus in higher molar solubilities. Consequently, the degree of asymmetry in fluid systems is defined by the *T_c_* and *P_c_* values of both the ILs and the compounds dissolved in them. In particular, the lower critical constants of the IL solvents and the higher critical constants of the solutes increase the system symmetries and thus—the solubilities. 

**Figure 1:**
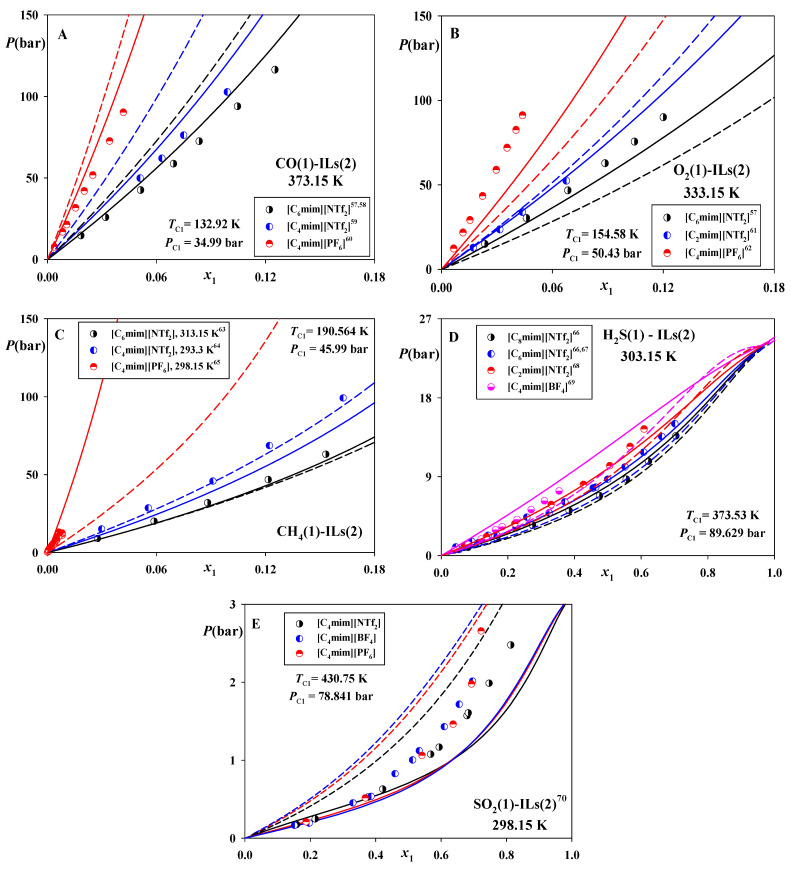
Solubilities of CO, O_2_, CH_4_, H_2_S and SO_2_ in [C_2_mim][NTf_2_], [C_4_mim][NTf_2_], [C_6_mim][NTf_2_], [C_8_mim][NTf_2_], [C_4_mim][BF_4_] and [C_4_mim][PF_6_]. Points—experimental data [57,58,59,60,61,62,63,64,65,66,67,68,69,70]. Solid lines—predictions of CP-PC-SAFT, dashed lines—predictions of SAFT-VR-Mie. *k*_12_ = 0 for both models. x1—mole fraction of the solute gas.

So far [41,42], a validity of this regularity was demonstrated for the IL systems of various non-associating compounds, such as refrigerants, aliphatic, aromatic, and oxygenated hydrocarbons. Figure 1 shows that the IL systems of CO, O_2_, CH_4_, H_2_S and SO_2_ also obey these regularities. As seen, the solvent capacities of the [NTf_2_]^−^ ILs are higher than of [PF_6_]^−^ and [BF_4_]^−^. Besides that, elongation of the cation *n*-alkyl chain also increases the solubilities, while the higher *T_c_* and *P_c_* values of the gases result in an increase of their solubilities. In can also be seen that both CP-PC-SAFT and SAFT-VR-Mie are, in general, capable of predicting these tendencies without adjusting the binary parameter *k*_12_. The only exception is the results of CP-PC-SAFT for the SO_2_ systems (Figure 1E), which do not exhibit any clear tendency. 

Nevertheless, this model predicts more accurately phase equilibria in the systems of CO (Figure 1A) and O_2_ (Figure 1B), along with solubility of CH_4_ in [C_4_mim][PF_6_] (Figure 1C). At the same time, SAFT-VR-Mie is advantageous in the case of its solubility in [C_4_mim][NTf_2_]. Besides that, accuracies of both models in predicting the solubilities of H_2_S in the considered ILs at 313.15 K are reasonably good (Figure 1D). CP-PC-SAFT tends to slightly underestimate them, while SAFT-VR-Mie minorly overpredicts these data.

**Figure 2:**
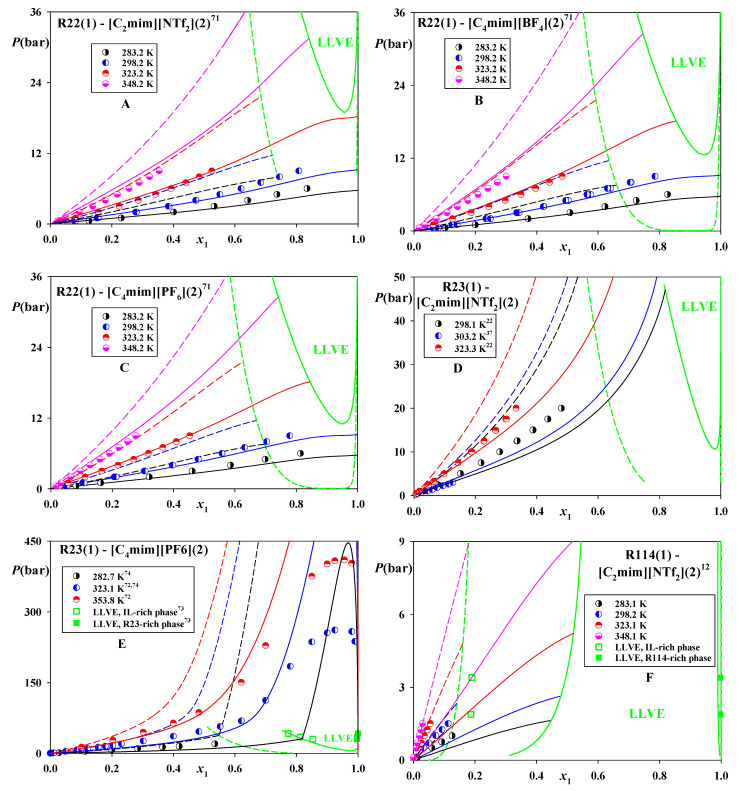
Phase equilibria in the systems of R22, R23 and R114 dissolved in [C_2_mim][NTf_2_], [C_4_mim][BF4] and [C_4_mim][PF_6_]. Points—experimental data [12,22,37,71,72,73,74]. Solid lines—predictions of CP-PC-SAFT, dashed lines—predictions of SAFT-VR-Mie. *k*_12_ = 0 for both models.

A success of CP-PC-SAFT and SAFT-VR-Mie in estimating solubility trends of refrigerants in the considered ILs was already discussed [42]. In the current study a more detailed comparison of their predictions for phase equilibria in these systems is presented. Figure 2A–C demonstrates that CP-PC-SAFT accurately predicts VLE in the systems of R22 and slightly underestimates them in R23—[C_2_mim][NTf_2_] (Figure 2D). At the same time, this model yields accurate results for VLE and LLVE in the system R23—[C_4_mim][PF_6_] and overestimates its LLE at high pressures (Figure 2E).

It can also be seen that SAFT-VR-Mie exhibits an inferior performance overestimating these data. At the same time, the wider phase splits yielded by this model result in more accurate in predictions of VLE and LLVE in R114—[C_2_mim][NTf_2_] (Figure 2F).

**Figure 3:**
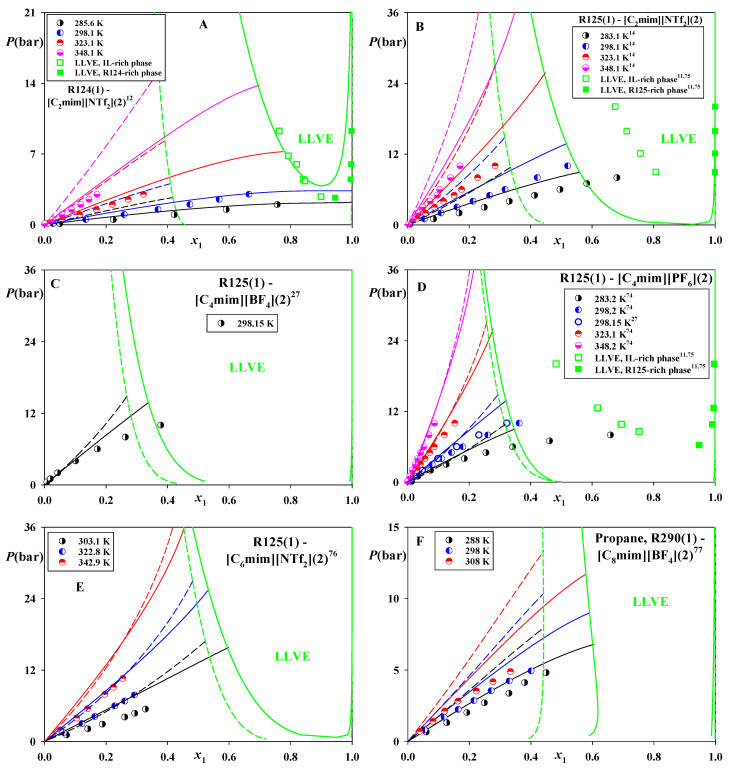
Phase equilibria in the systems of R124, R125, and propane (R290) dissolved in [C_2_mim][NTf_2_], [C_6_mim][NTf_2_], [C_4_mim][BF_4_], [C_8_mim][BF_4_] and [C_4_mim][PF_6_]. Points—experimental data [11,12,14,27,75,76,77]. Solid lines—predictions of CP-PC-SAFT, dashed lines—predictions of SAFT-VR-Mie. *K*_12_ = 0 for both models.

Figure 3 demonstrates that CP-PC-SAFT accurately predicts both the VLE and LLVE in R124–[C_2_mim][NTf_2_] (Figure 3A) and slightly overestimates VLE in systems of R125 and propane (Figure 3B–F). At the same, it overestimates the available LLVE data (Figure 3B,D) in a more significant manner. 

For some of the presented systems (see Figure 3A–C,F) SAFT-VR-Mie predicts substantially wider ranges of phase splits, which results in inferior accuracy in comparison with CP-PC-SAFT. For other systems (see Figure 3D,E), the results of both models are similar.

**Figure 4:**
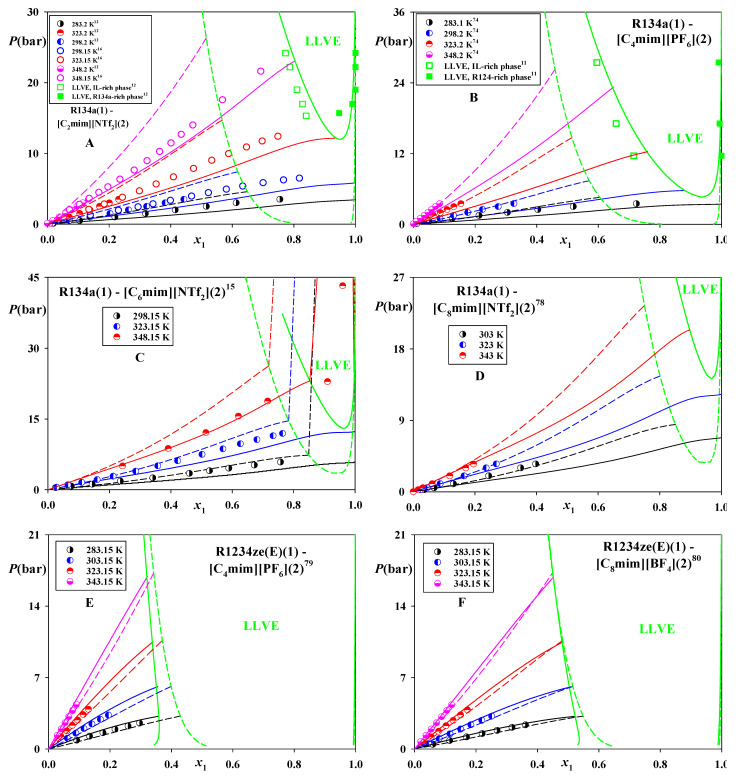
Phase equilibria in the systems of R134a and R1234ze(E) dissolved in [C_2_mim][NTf_2_], [C_6_mim][NTf_2_], [C_8_mim][NTf_2_], [C_8_mim][BF_4_], and [C_4_mim][PF_6_]. Points—experimental data [11,12,15,16,74,78,79,80]. Solid lines—predictions of CP-PC-SAFT, dashed lines—predictions of SAFT-VR-Mie. *K*_12_ = 0 for both models.

Figure 4A–D show that CP-PC-SAFT yields reasonably good predictions of VLE and LLVE in the systems of R134a, tending to slightly underestimate them. At the same time, it overestimates the high-pressure LLE in R134a—[C_6_mim][NTf_2_] at 348.15 K (Figure 4C). Once again, SAFT-VR-Mie yields broader ranges of phase equilibria for these systems, which usually deteriorates its accuracy. At the same time, in the case of R134a—[C_8_mim][NTf_2_] (Figure 4D) the performance of SAFT-VR-Mie is superior. Figure 4E,D demonstrate that both modes yield similarly accurate estimations for VLE in the systems R1234ze(E)—[C_4_mim][PF_6_] and—[C_8_mim][BF_4_]. Remarkable, unlike other considered cases, for these systems SAFT-VR-Mie predicts slightly narrower ranges of VLE phase splits.

**Figure 5:**
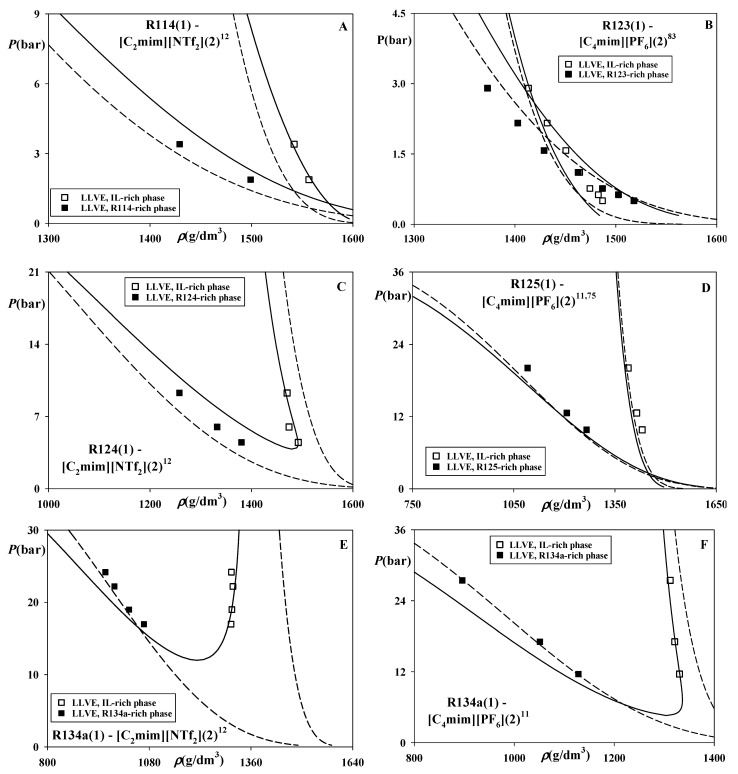
Densities of saturated liquid phases along LLVE in the systems of R114, R123, R124, R134a dissolved in [C_2_mim][NTf_2_] and [C_4_mim][PF_6_]. Points—experimental data [11,12,75,83]. Solid lines—predictions of CP-PC-SAFT, dashed lines—predictions of SAFT-VR-Mie. *k*_12_ = 0 for both models.

Figure 5 exemplifies predictions of the saturated liquid phases densities along LLVE. As seen, the results of both models in predicting these challenging data are usually reasonably good. Remarkable, despite the significant differences between CP-PC-SAFT and SAFT-VR-Mie in estimating the LLVE compositions of some systems (see Figure 2F, Figure 3A, Figure 4B), their results of for the densities can be rather similar (see Figure 5A,C,F). Moreover, despite the nearly identical overestimations of the LLVE phase splits in R125-[C_4_mim][PF_6_] (Figure 3D) by both models, the predictions of the pertinent densities are particularly accurate. At the same time, while CP-PC-SAFT accurately predicts both the compositions and the densities of R134a—[C_2_mim][NTf_2_] (Figure 4A and Figure 5E), a major overestimation of the LLVE range by SAFT-VR-Mie affects its estimations of the densities as well. Besides that, it can be seen that both models are capable of predicting the barotropy effect in the system R123—[C_4_mim][PF_6_] (Figure 3B). Remarkable, SAFT-VR-Mie estimates the barotropic point more accurately than CP-PC-SAFT. 

**Figure 6:**
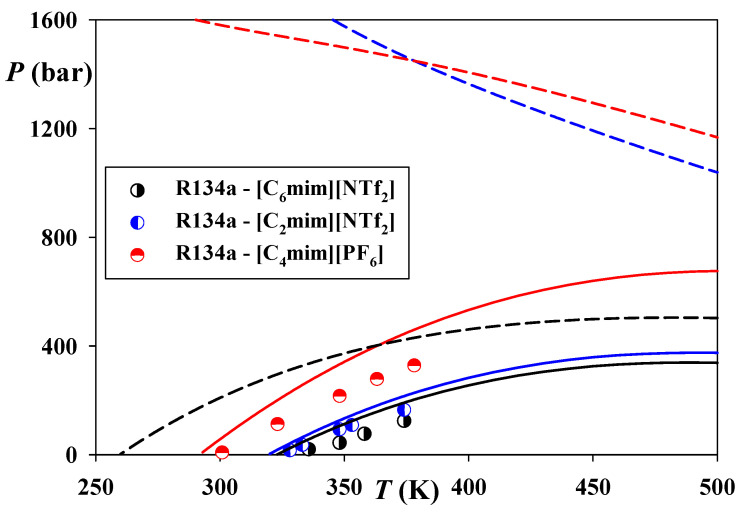
Critical loci in the systems of R134a and [C_6_mim][NTf_2_], [C_2_mim][NTf_2_], and [C_4_mim][PF_6_]. Points—experimental data [84]. Solid lines—predictions of CP-PC-SAFT, dashed lines—predictions of SAFT-VR-Mie. *k*_12_ = 0 for both models.

Figure 6 depicts the critical loci in the systems of R134a and [C_6_mim][NTf_2_], [C_2_mim][NTf_2_], and [C_4_mim][PF_6_]. Expectedly, the experimental critical pressures of these systems rise with a decrease of the Ils’ compressibilities and the corresponding increase of their systems asymmetry. All these systems exhibit Type V behavior according to the classification of van Konynenburg and Scott [81,82].

As seen, CP-PC-SAFT correctly predicts both the topology of all these systems and the tendencies established by their critical data. Certain overestimations of the critical pressures ire not surprising while bearing in mind the results of this model for the available LLE data (Figure 4C). At the same time, SAFT-VR-Mie overestimates the critical loci in a much more significant manner. Figure 6 demonstrates that it correctly predicts the phase behavior of Type V only for the most symmetric R134a—[C_6_mim][NTf_2_] system. 

For two more asymmetric systems of [C_2_mim][NTf_2_], and [C_4_mim][PF_6_], this model incorrectly estimates the behavior of Type III. This result also explains the shapes of LLVE and their densities yielded by SAFT-VR-Mie for these systems (see Figure 4A,B and Figure 5E,F), which do not exhibit a lower critical endpoint (LCEP). It can also be seen that SAFT-VR-Mie erroneously predicts the phase behavior of Type III for the system R124—[C_2_mim][NTf_2_], while CP-PC-SAFT correctly estimates for it, Type V (see Figure 3A and Figure 5C).

## 4. Conclusions

This study compared performances of two molecularly based approaches, namely CP-PC-SAFT and SAFT-VR-Mie, in predicting the available data on VLE, LLVE, critical loci and saturated phase densities of systems of CO, O_2_, CH_4_, H_2_S, SO_2_, propane, and the refrigerants R22, R23, R114, R124, R125, R125, R134a, and R1234ze(E) with [C_2_mim][NTf_2_], [C_4_mim][NTf_2_], [C_6_mim][NTf_2_], [C_8_mim][NTf_2_], [C_4_mim][BF_4_], [C_8_mim][BF_4_], and [C_4_mim][PF_6_] ILs. Both models were implemented to mixtures in an entirely predictive manner with *k*_12_ = 0. In order to preserve a predictive character of the pure compound parameterization, all the compounds were treated as non-polar and non-associating. This approach can be assessed as oversimplification because some of the considered compounds are polar and associating. At the same time, these intermolecular interactions influence the values of the critical constants, and therefore their effects are still indirectly counted. Remarkably, in the cases of polar and possibly associating compounds, SAFT-VR-Mie yields higher overestimations of the critical pressures. It has been demonstrated that the systems of CO, O_2_, CH_4_, H_2_S and SO_2_ obey a regularity previously detected for the systems of refrigerants and other compounds, namely a rise of solubilities with an increase of the ILs’ compressibilities. This regularity is explained by the fundamental GPD considerations. Since the imaginary *T_c_* and *P_c_* values of the more compressible ILs are lower, their systems are more symmetric, thus increasing the solubilities and vice versa. Similarly, the higher critical constants of solutes also reduce the asymmetry and increase the solubilities.

SAFT-VR-Mie is a more sophisticated model versus CP-PC-SAFT and its theoretical background is more advanced. Nevertheless, it appears that the GPD factors have a major influence on predicting phase equilibria in the IL systems. In particular, while CP-PC-SAFT rigorously obeys the literature values of both *T_c_* and *P_c_* of gases, SAFT-VR-Mie matches just the *T_c_* and overestimates the *P_c_*. The latter is supposed to reduce the systems asymmetry. Nevertheless, being fitted to the same high-pressure density data of the considered ILs, SAFT-VR-Mie yields the higher *T_c_* and *P_c_* values versus CP-PC-SAFT. Unfortunately, at the current level of expertise, it does not seem possible to judge which IL critical constants sets are more realistic. Nevertheless, it has been demonstrated that the higher critical constants of ILs generated by SAFT-VR-Mie probably result in overestimating the ranges of phase splits and lead to an inferior accuracy in predicting phase equilibria in many systems compared with CP-PC-SAFT. However, in certain cases, both models yielded similar results. Moreover, some data, such as VLE in the systems R114—[C_2_mim][NTf_2_] and R134a—[C_4_mim][PF_6_], along with the barotropic point of R123—[C_4_mim][PF_6_], were predicted more accurately by SAFT-VR-Mie. At the same time, despite a number of quantitative inaccuracies, both models were capable of reproducing the regularities characteristic for the considered systems, which makes them suitable for preliminary estimation of selectivity of the ILs in separating various gases.

## Figures and Tables

**Table 1:** Pure compound molecular parameters of CP-PC-SAFT and CS-SAFT-VR-Mie and overestimation of *P_c_* by CS-SAFT-VR-Mie (Δ*P*).

	CP-PC-SAFT			CS-SAFT-VR-Mie (*λ*_a_ = 6)				
Compound	*m*	*σ* [Å]	*ε*/*k*_b_ [K]	*m*	*λ*_r_	*σ* [Å]	*ε*/*k*_b_ [K]	Δ*P*
CO	0.9983	3.6437	99.474	1	21.38	3.6901	132.62	1.082
O_2_	1.0546	3.3055	112.98	1	17.95	3.4069	144.09	1.091
CH_4_	1.0001	3.7476	142.51	1	16.40	3.7532	170.81	1.096
H_2_S	1.2332	3.4229	254.04	1	26.69	3.7944	401.27	1.114
C_3_H_8_	2.4144	3.3918	184.37	2	11.82	3.6531	205.57	1.084
SO_2_	3.3034	2.5760	189.41	2	15.74	3.0908	287.98	1.136
R22	3.2452	2.8738	163.48	2	14.426	3.4262	234.54	1.130
R23	4.2360	2.4331	120.42	2	16.838	3.1792	207.36	1.191
R114	2.0403	4.1115	224.78	2	16.119	4.1764	283.70	1.084
R123	3.0540	3.4931	207.02	2	18.043	4.1005	328.26	1.119
R124	3.3472	3.2173	173.12	2	18.416	3.9345	287.09	1.104
R125	3.2728	3.0946	149.66	2	19.755	3.7421	254.23	1.124
R134a	3.9726	2.8502	154.00	2	21.627	3.6783	291.35	1.185
R1234ze(E)	2.9581	3.3370	175.47	2	21.342	3.8871	296.26	1.134

**Table 2:** The molecular parameters and critical constants of pure ILs yielded by SAFT-VR-Mie and CP-PC-SAFT [39,42].

	CP-PC-SAFT					SAFT-VR-Mie (*λ*_a_ = 6, *λ*_r_ = 12)				
Compound	*m*	*σ* (Å)	$\varepsilon /k_{B}(K)$	*T_c_* (K)	*P_c_* (bar)	*m*	*σ* (Å)	$\varepsilon /k_{B}(K)$	*T_c_* (K)	*P_c_* (bar)
[C_8_mim][NTf_2_]	*m* = −7.2461 + 0.0408 *M*_w_ *σ* = 3.5748 − 0.00012 *M*_w_ *ε*/*k*_b_ = 382.8867 − 0.2656 *M*_w_			834.2	10.32	9.3326	3.8650	343.69	1030.0	14.39
[C_6_mim][NTf_2_]				841.8	12.08	8.7544	3.8168	341.63	1007.0	15.94
[C_4_mim][NTf_2_]				846.1	14.28	8.1763	3.7685	351.58	1017.7	18.36
[C_2_mim][NTf_2_]				846.0	17.05	7.5981	3.7203	370.44	1051.1	21.76
[C_8_mim][BF_4_]	8.4287	3.5692	298.31	897.6	18.35	6.8632	3.8455	381.50	1051.8	22.60
[C_4_mim][PF_6_]	6.8113	3.5923	325.28	929.0	25.10	5.4343	3.8993	418.22	1074.9	30.26
[C_4_mim][BF_4_]	5.9300	3.6426	338.02	930.0	29.04	4.9271	3.8976	426.40	1062.2	34.09

## Data Availability

All the experimental data were obtained from the open sources and the relevant references were provided.

## Sample Availability

All the CP-PC-SAFT and SAFT-VR-Mie codes written in Mathematica software are available from the authors.
